# Modulators of arginine metabolism support cancer immunosurveillance

**DOI:** 10.1186/1471-2172-10-1

**Published:** 2009-01-09

**Authors:** Giusy Capuano, Nicolò Rigamonti, Matteo Grioni, Massimo Freschi, Matteo Bellone

**Affiliations:** 1 Cancer Immunotherapy and Gene Therapy Program, Istituto Scientifico San Raffaele, Via Olgettina 58, 20132, Milan, Italy; 2 Università Vita-Salute San Raffaele, Via Olgettina 58, 20132, Milan, Italy; 3 Unità Operativa Anatomia Patologica, Istituto Scientifico San Raffaele, Via Olgettina 58, 20132, Milan, Italy

## Abstract

**Background:**

Tumor-associated accrual of myeloid derived suppressor cells (MDSC) in the blood, lymphoid organs and tumor tissues may lead to perturbation of the arginine metabolism and impairment of the endogenous antitumor immunity. The objective of this study was to evaluate whether accumulation of MDSC occurred in Th2 prone BALB/c and Th1 biased C57BL/6 mice bearing the C26GM colon carcinoma and RMA T lymphoma, respectively, and to investigate whether N(G) nitro-L-arginine methyl ester (L-NAME) and sildenafil, both modulators of the arginine metabolism, restored antitumor immunity.

**Results:**

We report here that MDSC accumulate in the spleen and blood of mice irrespective of the mouse and tumor model used. Treatment of tumor-bearing mice with either the phosphodiesterase-5 inhibitor sildenafil or the nitric-oxide synthase (NOS) inhibitor L-NAME significantly restrained tumor growth and expanded the tumor-specific immune response.

**Conclusion:**

Our data emphasize the role of MDSC in modulating the endogenous tumor-specific immune response and underline the anti-neoplastic therapeutic potential of arginine metabolism modulators.

## Background

An established tumor adopts several strategies to escape immunosurveillance and this complex phenomenon results in generation of a site of acquired immune privilege [1]. Over time, local suppression spreads systemically, thereby weakening immunological barriers that might protect against tumor metastasis. Tumor-specific suppression might explain why even immunotherapies that succeed in inducing systemic immune response are rarely of clinical effect on tumors.

As reviewed in [2], impairment in tumor antigen expression or its processing and presentation by both tumor cells and antigen presenting cells (APC), release of immunouppressive cytokines and prostaglandins as well as pro-apoptotic mechanisms may directly and/or indirectly impair T cell function while favoring tumor cell growth. Finally, tumor cells may promote development and recruitment of regulatory T cells (Treg) and myeloid derived suppressor cells (MDSC). CD4^+^CD25^+ ^Treg in particular, represent an essential mechanism of peripheral tolerance to self antigens [3]. They selectively express Foxp3, a forkhead/winged helix transcription factor that controls master genes in Treg development/function [3]. Several neoplasms associate with CD4^+^CD25^+ ^Treg accumulation in the blood and/or in tumors, and this may inversely correlate with patients' survival [4].

MDSC are a heterogeneous population of cells of myeloid origin [5], and include immature macrophages, granulocytes, dendritic cells (DC) and other myeloid cells [2,5-7]. Whereas in the spleen of normal mice they account for less than 5% of the nucleated cells, they rapidly accumulate in secondary lymphoid organs, blood and tissues during inflammation and cancer [6,8]. Several soluble factors contribute to alteration of the normal myelopoiesis and recruitment of MDSC to peripheral organs under pathologic conditions, including IL-3, IL-6, IL-10, vascular endothelial growth factor (VEGF), macrophage colony-stimulating factor (M-CSF) and granulocyte-macrophage colony-stimulating factor (GM-CSF) [6-8]. In mice, MDSC are characteristically CD11b^+^Gr-1^+^, and may also express CD31 [9], CD124, IL-4 receptor α-chain [10] and CD80 [11]. Expression of CD115 on MDSC may correlate with their ability to mediate development of Treg [12]. In humans MDSC have been described to accumulate in the peripheral blood of patients affected by breast, lung, renal and head and neck carcinomas [6] and in melanoma [13], but their phenotype is still poorly defined. MDSC impair T lymphocyte functions through different mechanisms, including immunosuppressive cytokines and perturbation of the arginine metabolism by inducible nitric oxide synthase (iNOS), arginase (Arg), and reactive oxygen species [14]. More in details, iNOS produces nitric oxide (NO), which interferes with IL-2 receptor signaling [15], leading to cell cycle arrest. NO is also a key signaling molecule in inflammation-driven diseases, including cancer, where it participates to cancerogenesis, angiogenesis, tumor cell proliferation and invasion [16]. Furthermore, high Arg activity depletes arginine from the microenvironment, inhibiting T cell activation and proliferation [17], and favoring T cell apoptosis [14].

Several Arg and NOS inhibitors have been tested with the purpose to inhibit tumor development and favor antitumor immunity [18]. As an example, N(G)-monomethyl-L-arginine as been shown to restore anti-tumor immunity *in vitro *[19]. Unfortunately, its use in clinic has been discontinued due to severe toxicity [18]. N(G) nitro-L-arginine methyl ester (L-NAME) has been reported in several mouse models to inhibit tumor growth [20-22]. Those studies however, did not investigate a direct correlation of its effects on the endogenous tumor-specific immune response. More recently, Serafini et al. [23] reported that phosphodiesterase-5 inhibitors (sildenafil, tadalafil and vardenafil) down regulate Arg and iNOS expression, thereby impairing the immunosuppressive activity of MDSC. In the mouse models tested, restoration of T cell immunity correlate with substantial delay in tumor progression [23].

We evaluated whether accumulation of MDSC occurred in BALB/c and C57BL/6 mice bearing the C26GM colon carcinoma and RMA T lymphoma, respectively, and we investigated whether L-NAME and sildenafil restored antitumor immunity and delayed tumor growth.

## Results and Discussion

### MDSC accumulate in the spleen and blood of tumor-bearing mice

**Figure 1:**
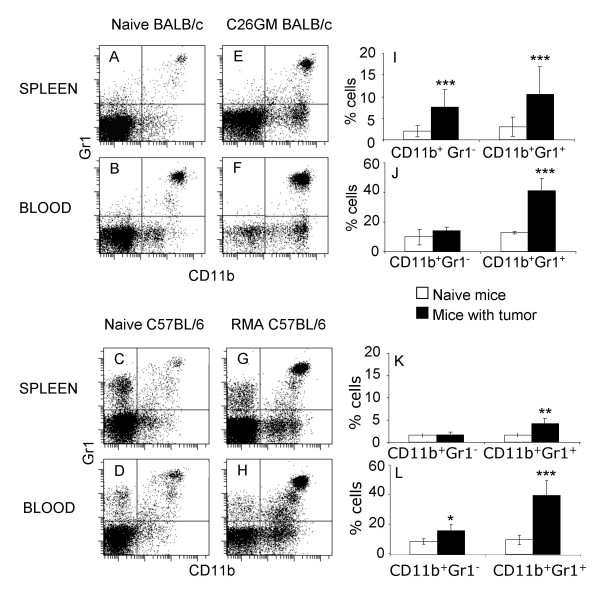
**Tumor-bearing mice have an expanded pool of MDSC in spleen and blood**. Splenocytes and blood cells from naïve (A-D) and tumor bearing BALB/c (E, F) and C57BL/6 mice (G, H) were stained for CD11b and Gr1 markers and analyzed by flow cytometry as detailed in the Materials and Methods section. Dot plot panels are representative of 8 independent experiments for a total 10 naïve (A, B) and 38 tumor-bearing BALB/c mice (E, F) and 15 naive (C, D) and 15 RMA-bearing C57BL/6 mice (G, H), respectively. Histograms report the percentage of CD11b^+ ^Gr1^- ^and CD11b^+ ^Gr1^+ ^cells as aggregated data (average ± SD) for the BALB/c (I, J) and the C57BL/6 models (K, L), respectively. Statistic analysis of collected data was performed using the Student's T test; ***p < 0.001, **0.001 < p < 0.05.

We initially investigated the effect of tumor growth on myelopoiesis and recruitment of MDSC to peripheral organs in the well-characterized colon adenocarinoma C26GM model [24]. This is a more aggressive variant of the carcinogen-induced undifferentiated colon carcinoma CT26 [25], genetically modified to secrete GM-CSF [24]. C26GM is particularly interesting owing to its aggressiveness (animals are killed by the tumor in less than 2 weeks) and because of the secretion of GM-CSF, one of the factors known to alter myleopoiesis during tumor growth [7]. Hence, C26GM cells were injected s.c. in BALB/c mice, and animals were killed 9 days later, when the tumor mass had reached the dimension of approximately 600 mm^3^. Flow cytometry analysis of the spleen and blood of tumor-bearing mice showed a dramatic accumulation of CD11b^+^Gr1^+ ^cells in tumor-bearing mice (Fig. 1E and 1F, respectively) when compared with naïve age- and sex-matched littermates (Fig. 1A and 1B). Quantification of CD11b^+^Gr1^+ ^cells demonstrated a statistically significant increase in both the spleen and blood of tumor-bearing mice (Fig. 1I and 1J). Interestingly, a second population of CD11b^+^Gr1^- ^cells, barely detectable in the spleen of naïve BALB/c mice, became well represented in tumor-bearing mice, therefore confirming previous reports [24]. CD11b^+^Gr1^- ^cells were already detectable in the blood of naïve BALB/c mice, and marginally increased during tumor growth (Fig. 1B, F and 1J). It has been reported that both populations suppress CD8^+ ^T lymphocytes [10], suggesting that Gr1^+ ^and Gr1^- ^fractions of CD11b^+ ^cells are differentiation steps of the same MDSC population. Indeed, CD11b^+^Gr1^+ ^cells may differentiate both *in vitro *and *in vivo *into Gr1^- ^cells [26-28].

**Figure 2:**
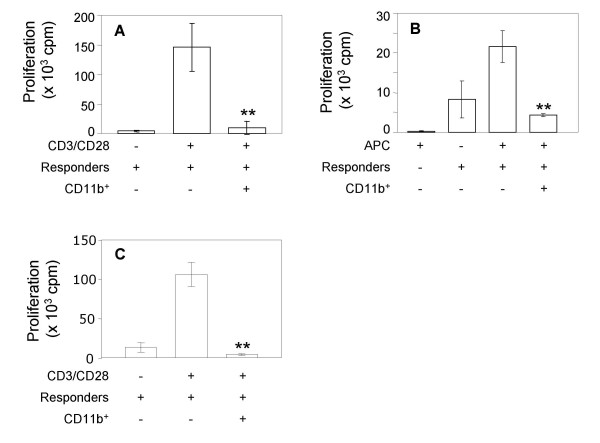
**MDSC suppress T cells proliferation**. MDSC were purified as CD11b^+ ^cells from the spleen of C26GM (A, B) and RMA (C) tumor bearing mice by immunomagnetic beads sorting as detailed in the Materials and Methods section. CD11b^+ ^cells were added at a final concentration of 20% to a mixed leukocyte culture set up with splenocytes, as responders, stimulated with anti-CD3 and anti-CD28 antibodies (A, C) or with an equal number of γ-irradiated C57BL/6 splenocytes (B). Data are representative of at least two experiments and are expressed as the cpm mean ± SD of triplicates. Student's T test: **0.001 < p < 0.05.

To verify whether the CD11b^+ ^cells accumulating in tumor-bearing BALB/c mice were "bona fide" MDSC, splenic CD11b^+ ^cells were purified by magnetic bead sorting, and tested for their immunosuppressive activity in standard *in vitro *proliferation assays. Indeed, CD11b^+ ^cells purified from C26GM tumor-bearing mice were able to substantially abrogate the proliferation of BALB/c splenocytes stimulated with either anti-CD3 and anti-CD28 antibodies (Fig. 2A) or γ-irradiated C57BL/6 splenocytes (Fig. 2B).

The immune response to pathogens and tumors is genetically controlled, and may diverge in different mouse strains. As an example, BALB/c mice are considered a Th2 prone strain, whereas Th1 responses dominate in C57BL/6 mice. Because Th2-associated cytokines (e.g. IL-6) and other soluble factors are needed to alter normal myelopoiesis and favor recruitment of MDSC to peripheral organs [6-8], and both Th1 (IFN-γ) and Th2 (IL-13) cytokines are required for activation of MDSC [10], a balance between Th1 and Th2 immune responses may be required for MDSC accumulation and immunosuppressive function during tumor progression. To investigate whether similar populations of MDSC accumulated also in a Th1-prone strain upon tumor challenge, C57BL/6 mice were implanted s.c. with RMA cells, a Rauscher virus-induced T lymphoma [29]. Because RMA is less aggressive than C26GM, mice were killed 2 weeks after tumor challenge, time at which the mass had reached a dimension of approximately 400 mm^3^. Tumor-associated accumulation of CD11b^+^Gr1^+ ^cells in the blood of C57BL/6 mice was comparable to the one found in tumor-bearing BALB/c mice (Fig. 1D, H and 1L and 1B, F and 1J, respectively). A less dramatic but still statistical significant accumulation of CD11b^+^Gr1^+ ^cells was evident also in the spleen of mice bearing RMA lymphomas (Fig. 1C, G and 1K). Accumulation of CD11b^+^Gr1^- ^cells was less conspicuous in C57BL/6 mice than in tumor-bearing BALB/c mice (Fig. 1K and 1L and 1I and 1J, respectively), and was statistically significant only when blood samples from naïve and tumor-bearing mice were compared (Fig. 1L). Nevertheless, CD11b^+ ^cells purified from RMA tumor-bearing mice inhibited proliferation of syngenic splenocytes to an extent comparable to CD11b^+ ^cells purified from C26GM tumor-bearing mice (Fig. 2C).

Hence, tumor-induced altered regulation of myelopoiesis and accumulation of MDSC is a characteristic common to Th1 and Th2 strains as well as to tumors of different aggressiveness. Quantitative and qualitative differences in MDSC populations found in the two models may depend on several factors, among which the genetic background, tumor aggressiveness, and soluble factors released by tumor and stroma cells, including bone marrow derived cells, fibroblasts and endothelial cells [30].

### Treatment of tumor-bearing mice with Arg and iNOS inhibitors restrain tumor growth

**Figure 3:**
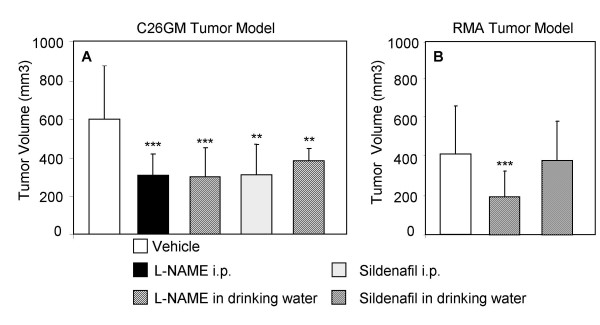
**Modulators of arginine metabolism restrain tumor growth**. BALB/c (A) and C57BL/6 mice (B) were challenged s.c. with C26GM and RMA cells, respectively. The same day mice were randomly assigned to either one of the following treatments: L-NAME or sildenafil (given i.p. or dissolved in drinking water) or vehicle (PBS) i.p. (see Materials and Methods section for experimental details). Tumor volume (expressed as mm^3^) was measured by caliper at day 9 and 15, respectively. Panels report the cumulated data of at least three independent experiments performed with the following number of animals: C26GM model: Vehicle: n = 24, L-NAME i.p.: n = 15, L-NAME o.s.: n = 14, Sildenafil i.p.: n = 10, Sildenafil o.s.: n = 5; RMA model: Vehicle: n = 15, L-NAME o.s.: n = 15, Sildenafil o.s.: n = 10. Student's T test: ***p < 0.001, **0.001 < p < 0.05.

Accumulation of MDSC in both BALB/c- and RMA-bearing mice suggested that alteration of arginine metabolism [31] was a relevant mechanism of immunoescape in these models, and that drugs able to modulate arginine metabolism [18] were potentially useful in crippling the immunosuppressive activity of MDSC and delaying tumor growth. To verify this hypothesis, BALB/c mice were challenged s.c. with C26GM cells, and L-NAME or sildenafil were administered starting on the day of tumor challenge. To identify the best delivery strategy, drugs were administered either i.p. or in the drinking water. As control, animals were treated with vehicle (i.e., PBS) only. Both inhibitors significantly delayed tumor growth by approximately 50% when compared with vehicle-treated mice, and both regimens were equally effective (Fig. 3A).

Similar experiments were conducted in the RMA model. Having verified in the C26GM model that the two delivery strategies were equally effective, and to avoid distress and/or pain to the animals, drugs were administered in the drinking water. Also in this model, L-NAME delayed tumor growth by approximately 50% (Fig. 3B). Conversely, sildenafil did not appear to be effective against RMA lymphoma. The scarce efficacy of sildenafil in restraining RMA growth does not appear to be related to the mouse strain, because Serafini et al. reported an excellent effect of sildenafil in C57BL/6 mice challenged with the fibrosarcoma MCA203 [23]. This discrepancy may more likely be related to the characteristics of the two tumor models (e.g., cytokine production, immunogenicity, aggressiveness, stromal reaction).

**Figure 4:**
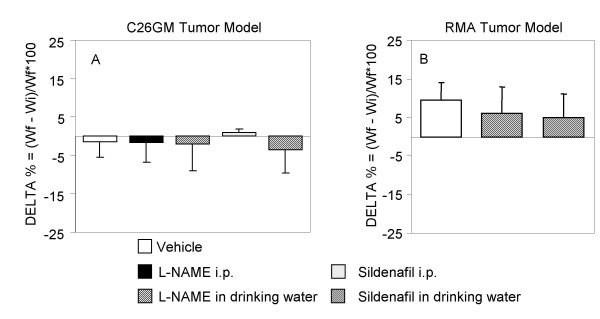
**Modulators of arginine metabolism do not cause loss of body weight in treated animals**. Toxicity of L-NAME and sildenafil was evaluated as loss of body weight, defined as Delta % = (final weight – initial weight)/initial weight × 100. BALB/c mice challenged with C26GM cells (A) and C57BL/6 mice challenged with RMA cells (B) were weighed at day 0 and at day 9 and 15, respectively. The experimental groups are described in the legend to Fig. 3.

Since chronic treatment with NO inhibitors has been reported to be associated with systemic toxicity [32], we monitored animal weight, as a measure of potential drug-related toxicity, and we found no difference between drug- and vehicle-treated mice in both strains (Fig. 4A and 4B). The respiratory apparatus (i.e., bronchial tree and lung), esophagus, kidney, suprarenal gland and liver of L-NAME treated animals were also macroscopically and microscopically investigated by an expert pathologist and found with no sign of drug-related toxicity (data not shown). Absence of drug-related toxicity in our animal models might be related to the relatively short period of treatment. Indeed, glomerulosclerosis, one of the effects of L-NAME, is usually found in animals treated for a longer period of time [33].

### Modification of tumor-associated myelopoiesis induced by modulators of arginine metabolism

**Figure 5:**
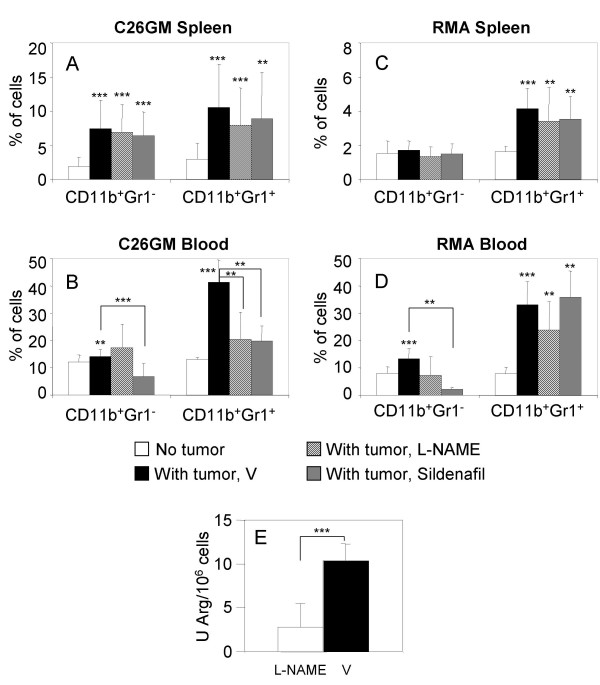
**Modulators of arginine metabolism alter MDSC accumulation in the blood of tumor-bearing mice**. BALB/c and C57BL/6 mice were challenged s.c. with C26GM and RMA cells, respectively. The same day, mice were randomly assigned to either one of the following treatments: L-NAME or sildenafil (given i.p. or dissolved in drinking water) or vehicle (PBS; V) i.p. (see Materials and Methods section for experimental details). Tumor-bearing BALB/c and C57BL/6 mice were killed 9 and 15 day later. Recruitment of CD11b^+^Gr1^- ^and CD11b^+^Gr1^+ ^cells in the spleen (A, C) and blood (B, D) of tumor-bearing BALB/c (A, B) and C57BL/6 mice (C, D) was investigated by flow cytometry. Histograms report the percentage of CD11b^+ ^Gr1^- ^and CD11b^+ ^Gr1^+ ^cells as aggregated data (average ± SD) for the experimental groups reported in the legend of Fig. 3. Alternatively, magnetic bead sorted CD11b^+ ^cells from the spleen of RMA (E) tumor bearing mice were lysed and analyzed for relative Arg enzyme activity. Student's T test: ***p < 0.001, **0.001 < p < 0.05.

We investigated whether treatment with L-NAME or sildenafil modified accrual of MDSC in the spleen and blood of tumor-challenged mice. Flow cytometry analyses conducted *ex vivo *the day of mouse killing showed a clear accumulation of both CD11b^+^Gr1^+ ^and CD11b^+^Gr1^- ^cells in the spleen of BALB/c mice bearing C26GM tumors, but neither L-NAME nor sildenafil altered such recruitment (Fig 5A). Conversely, CD11b^+^Gr1^+ ^cells dropped by 50% in the blood of tumor-bearing mice treated with either L-NAME or sildenafil, when compared with samples collected from untreated tumor-bearing mice (Fig. 5B). This was not confirmed in the RMA model, despite a comparable recruitment of CD11b^+^Gr1^+ ^cells (Fig. 5D). Hence, L-NAME, although highly effective in restraining tumor growth in both models, do not appear to consistently perturb the tumor-associated recruitment of CD11b^+^Gr1^+ ^cells. To gain further insight on the mechanism underlying the therapeutic effect of L-NAME, we assessed the enzymatic activity of MDSC cells purified from the spleen of RMA-bearing mice. Magnetic bead-purified CD11b^+ ^cells from L-NAME treated animals showed a relevant reduction in Arg activity when compared with those obtained from control animals treated with vehicle (Fig. 5E), therefore confirming that in vivo L-NAME is a potent inhibitor of Arg [34].

In both tumor models a reduction statistically significant of CD11b^+^Gr1^- ^cells was evident in the blood of sildenafil-treated mice when compared with untreated ones (Fig. 5B and 5D). Hence, it might be possible that sildenafil acts primarily on terminally differentiated CD11b^+^Gr1^- ^cells [27]. Together with the notion that *in vivo *sildenafil treatment reduces the enzymatic activity of iNOS and Arg in MDSC [23], our findings support the hypothesis that sildenafil has a potent inhibitory impact on the MDSC-mediated immunosuppressive mechanisms. Because sildenafil caused delayed tumor growth and a significant fall of both MDSC populations only in the C26GM model, it might be possible that sildenafil-induced containment of tumor growth is obtained only when myelopoiesis is redirected to more physiologic conditions. These hypotheses need further experimental support.

### Treatment with L-NAME does not perturb tumor-draining lymph node cell composition

**Figure 6:**
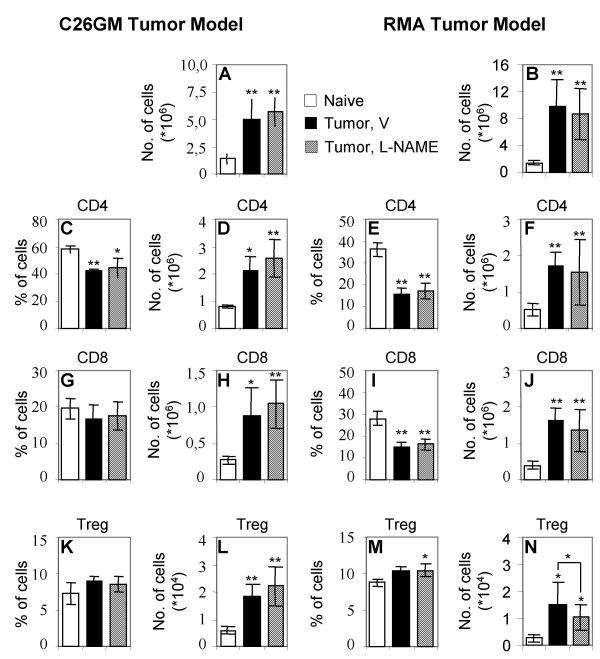
**Treatment of tumor-bearing mice with L-NAME does not perturb TDLN cell composition**. Cells from TDLN of C26GM- and RMA-bearing mice treated with L-NAME or vehicle (V) and from LN of naïve strain-related mice were either enumerated (A, B) or stained with T cell markers and analyzed by flow cytometry. Histograms report the percentage and absolute number of CD4^+ ^(C, E, and D, F), CD8^+ ^(G, I, and H, J), and CD4^+^CD25^+^Foxp3^+ ^T cells (Terg; K, M, and L, N), respectively, as aggregated data (average ± SD) for the experimental groups reported in the legend of Fig. 3. Student's T test: **0.001 < p < 0.05, *p < 0.05.

Lymph nodes draining a tumor mass (TDLN) or the site of vaccination rapidly enlarge owing to the accumulation of myeloid and lymphoid cells [35,36]. We asked whether arginine metabolism inhibitors could alter recruitment of T lymphocytes in TDLN. Because L-NAME demonstrated to be effective in restraining tumor growth in both models, we focused on this inhibitor. Hence, BALB/c mice were challenged with C26GM cells and treated with L-NAME or vehicle as described above. A 3-fold increase in cell number was evident in TDLN when compared with LN from naïve mice, and was not modified by L-NAME treatment (Fig. 6A). Flow cytometry analysis of TDLN cells showed a considerable increase in the number of both CD4 and CD8 cells (Fig. 6D and 6H, respectively). Curiously enough, the percentage of CD8 and more evidently of CD4^+ ^T cells in TDLN decreased in both L-NAME and vehicle-treated mice (Fig. 6G and 6C, respectively), suggesting that in TDLN a relevant perturbation of the physiologic equilibrium among the different cell populations was undergoing, that was not modified by L-NAME.

We recently reported that Treg accumulate in TDLN as well as in LN draining the site of vaccination [36], therefore suggesting that such recruitment is a physiologic hallmark of sites were an active immune response is taking place. Because MDSC can favor induction of Treg [12], we also investigated whether Treg recruitment in TDLN of BALB/c mice bearing C26GM tumors was further increased by tumor-associated MDSC and modified by L-NAME treatment. The absolute number of CD4^+^CD25^+^Foxp3^+ ^cells dramatically increased in TDLN, but was not modified by L-NAME treatment (Fig. 6L).

When similar analyses were conducted in TDLN collected from RMA-bearing C57BL/6 animals, results substantially overlapped the ones obtained in BALB/c mice bearing C26GM tumors. Indeed, no substantial differences in percentage and absolute number of CD4 and CD8 T cells were found in L-NAME and vehicle-treated mice (Fig. 6F and 6J, respectively). A slightly and yet statistically significant reduction in the absolute number of Treg was found in TDLN from L-NAME treated animals (Fig. 6N). A similar result was obtained in sildenafil treated animals (absolute number of Treg: 10,00 ± 0,24 × 10^3^; n = 5).

All together, these data suggest that L-NAME does not substantially modify the physiologic perturbation of LN composition in TDLN.

### Treatment with L-NAME augments the endogenous antitumor immunity

**Figure 7:**
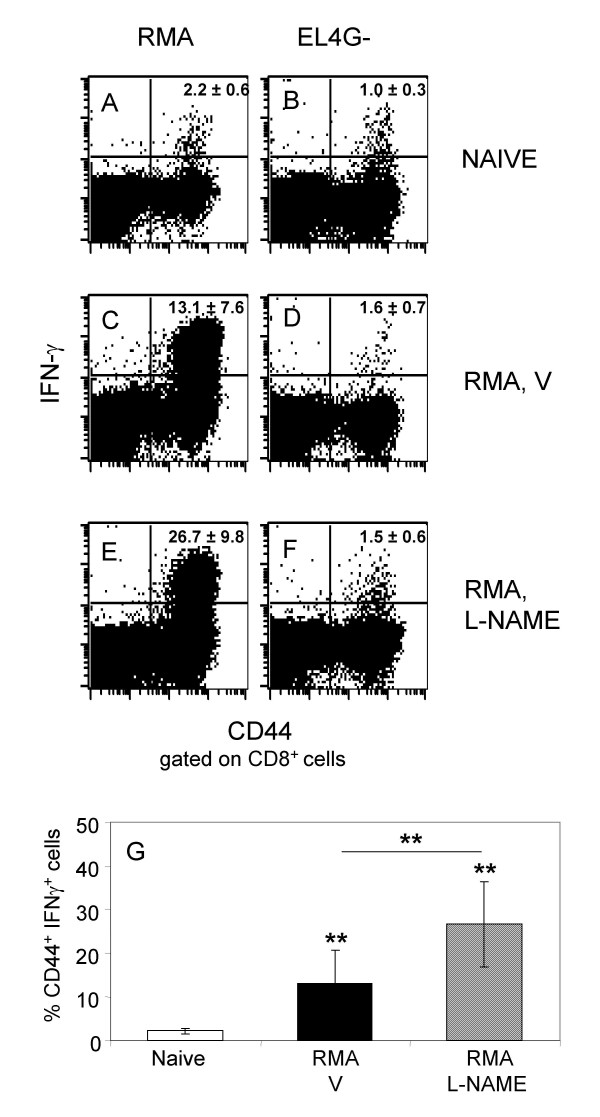
**Treatments with L-NAME improves IFN-γ release**. C57BL/6 mice were challenged with RMA cells and treated with L-NAME (E, F) or vehicle (V; panels C and D). After 15 days, animals were killed and their splenocytes were specifically restimulated *in vitro*. Blasts were tested for IFN-γ production (upon challenge with RMA or EL4G-) and analyzed by FACScalibur^® ^after co-staining with mAb against CD8, CD44 and IFN-γ. Panel A and B report representative dot plot analyses of blasts from cultures of naïve splenocytes that were challenged with RMA (A) or EL4G- targets (B). The percentage of CD8^+^IFN-γ^+ ^cells is reported in each panel as average ± SD of at least five animals for experimental group. The same data is reported in histogram G together with the statistical analysis. Student's T test: **0.001 < p < 0.05.

Our final goal was to investigate the effects of L-NAME on the endogenous tumor-specific immune response. RMA is an immunogenic tumor (i.e. upon *in vivo *growth it spontaneously induces an endogenous tumor-specific immune response [37]), whose immunogenicity is strongly biased by the expression of dominant viral antigens [38]. Hence, splenocytes recovered from L-NAME and vehicle-treated C57BL/6 mice bearing RMA tumors, were specifically restimulated *in vitro *and tested for their ability to recognize different targets. Flow cytometry analyses showed that cultures from L-NAME-treated mice contained a frequency of CD8^+ ^T cells producing IFN-γ upon RMA challenge (Fig. 7E) higher than the one found in cultures from vehicle-treated mice (Fig. 7C). IFN-γ release was specific because the percentage of cells challenged with the irrelevant target EL4G- was at background level in both cultures (Fig. 7F and 7D). RMA-specific response was not due to a *in vitro *priming, because cells from naïve animals did not produce IFN-γ above background level when challenged with the targets (Fig. 7A and 7B).

Since it is well known that NO participates in the regulation of various cell activities, to exclude that L-NAME and sildenafil exerted a direct effect on RMA cells by affecting doubling time and/or favoring apoptosis, we assessed the *in vitro *proliferation potential of RMA cells in the presence of either one of two drugs. Both L-NAME and sildenafil neither altered RMA proliferation rate, nor increased apoptosis (data not shown), therefore excluding this possibility.

Taken together, these data demonstrate that L-NAME treatment has the potential to augment the endogenous antitumor immunity. Because MDSC seem not to block the early events of T cell activation and IFN-γ production [10], we can hypothesize that L-NAME favors IFN-γ production by other yet uncovered mechanisms.

## Conclusion

Elucidation of the mechanisms by which a growing tumor eludes immunosurveillance and identification of strategies to overcome tumor-associated immunosuppression are essential issues that need to be addressed, in order to obtain the most rewarding clinical benefits from the application of immunotherapeutic strategies to cancer patients. Our results, obtained in different tumor models and murine strains, indicate that tumor growth is associated with perturbation of myelopoiesis and accumulation of MDSC. Treatment of tumor-bearing mice with modulators of arginine metabolism may modify such accrual, augment the endogenous tumor-specific immune response and restrain tumor growth.

Because neither in our nor in other tumor models inhibition of MDSC function by different means caused complete tumor regression [21,23,39,40], it is likely that acting simultaneously on more than one of the know tumor-associated immunosuppressive mechanisms will result in more successful therapeutic effects. As an example, sildenafil treatment improves the efficacy of adoptive cell therapy [23]. Further investigation in this direction is warranted to confirm this hypothesis.

## Methods

### Cell lines and reagents

C26GM (H-2^d^) is a carcinogen-induced colon carcinoma subcloned from the CT26 cell line, and genetically modified to produce GM-CSF [24]. Cells were cultured in DMEM (Invitrogen, Milano, Italy) supplemented with 2 mM L-glutamine, 150 U/ml streptomycin, 200 U/ml penicillin and 10% heat-inactivated FBS (Invitrogen). RMA (H-2^b^) is a Rauscher virus-induced thymoma [29]. EL4G^- ^(H-2^b^) is a 9,10-dimethyl-1,2-benzanthracene induced thymoma [41]. Both cell lines were cultured in RPMI-1640 (Invitrogen) supplemented with 2 mM L-glutamine, 150 U/ml streptomycin, 200 U/ml penicillin and 10% heat-inactivated FBS (Invitrogen). Unless specified, all chemical reagents were from Sigma-Aldrich, and monoclonal antibodies (mAb) were from BD PharMingen (San Diego, CA).

### Mice and *in vivo *experiments

BALB/c (H-2^d^) and C57BL/6 (H-2^b^) female mice, 8–10 weeks old, were purchased from Charles River (Calco, Italy), housed in a specific pathogen-free animal facility, and treated in accordance with the European Union guidelines, and with the approval of the Institutional Ethical Committee (IACUC approval # 311). Five hundred thousand C26GM cells and 7 × 10^4 ^RMA cells were injected s.c. on the left flank of BALB/c and C57BL/6 mice, respectively. L-NAME (given i.p. at 80 mg/Kg/24 h or added in drinking water at 1 g/L) or sildenafil (Pfizer, New York, NY, given i.p. at 20 mg/Kg/24 h or dissolved in drinking water at 20 mg/Kg/24 h) were administered starting on the day of tumor challenge. As control, groups of animals were treated with vehicle (PBS) only. Water was given *ad libitum*. Calculation of the dosage of each drug was based on the assumption that a mouse drinks approximately 3 ml/24 h. Tumor size was evaluated by measuring two perpendicular diameters by a caliper every other day, and BALB/c and C57BL/6 mice were sacrificed after 9 and 15 days, respectively. When needed, several organs were collected, fixed in 4% formalin for 6 h, then embedded and included in paraffin wax as previously described [42]. Five-mm thick sections were cut and stained with H&E (Bio-Optica, Milano, Italy).

### Phenotypic characterization of cell populations

TDLN and LN from naïve mice and tumor-challenged mice were processed on a cell strainer and stained with FITC-labeled anti-CD4, PE- labeled anti-CD44 and PerCP-Cy 5.5-labeled anti-CD8 mAb. Dead cells were excluded by physical parameters and/or by the addition of ToPro-5 (Molecular Probes, Eugene, OR) immediately before flow cytometry analysis. For enumeration of MDSC cells, blood and splenocytes from naïve and tumor-challenged mice were stained with FITC-conjugated CD11b and APC-conjugate Gr1 mAb. For enumeration of CD4^+^CD25^+^Foxp3^+ ^cells, LN cells were stained with FITC-labeled anti-CD4, PerCP-Cy 5.5-labeled anti-CD8 and APC-labeled anti-CD25 (clone PC61) mAb, permeabilized and finally, stained with PE-labeled anti-Foxp3 mAb (eBioscience, SanDiego, CA) according to the manufacturer's instructions. In all experiments, cells were analyzed on a BD FacsCalibur^®^.

### Intracellular cytokine measurement

Single cell suspensions of splenocytes from naïve and RMA-challenged mice were restimulated *in vitro *with mitomycin-c treated RMA cells [37,38]. For intracellular cytokine measurement, day-5-blasts were challenged *in vitro *with RMA or EL4G^- ^cells (1:1 ratio), or phorbol 12-myristate 13-acete (PMA)/ionomycin, and stained with FITC-labeled anti-CD44, PE-labelled anti-CD4, PerCP-Cy 5.5-labeled anti-CD8, and APC-labeled anti-IFN-γ mAb as previously described [43].

### CD11b^+ ^cell purification and in vitro functional assays

CD11b^+ ^cells purification was performed with mouse CD11b MicroBeads (Miltenyi Biotec, Bergisch Gladbach, Germany) following the manufacturer's instructions. Purity of the cell population was evaluated by flow cytometry and exceeded 90%. BALB/c splenocytes (6 × 10^5 ^cells/well) were stimulated in wells that had been coated with 3 μg/ml anti CD3 and 2 μg/ml anti-CD28 mAb, or alternatively, with an equal number of γ-irradiated C57BL/6 splenocytes. Purified splenic CD11b^+ ^cells were added to the culture so as to constitute 20% of the total cells. After 3 days of incubation, cultures were pulsed with ^3^H-Thymidine (1 μCi/well; Amersham Corp., Milan, Italy) for the last 18 h. The incorporation of ^3^H-Thymidine by proliferating T cells (triplicate cultures) was measured by scintillation counting. Alternatively, CD11b^+ ^cells were assessed for enzymatic activity by QuantiChrom™ Arginase Assay Kit (BioAssay Systems, Hayward, CA) following manufacturer's instructions. Results were normalized to 10^6 ^cells.

### Statistical analyses

Statistical analyses were performed using the Student's T test. Values were considered statically significant for p < 0.05.

## Authors' contributions

MB drafted the manuscript, all authors contributed to the revision. GC, NR, MF and MG performed experiments. MB and GC were involved in conception and design of the study. All authors read and approved the final manuscript.
